# TRAP1 inhibits MIC60 ubiquitination to mitigate the injury of cardiomyocytes and protect mitochondria in extracellular acidosis

**DOI:** 10.1038/s41420-021-00786-5

**Published:** 2021-12-14

**Authors:** Lingxiao Zhang, Ning Su, Yuanyuan Luo, Siyin Chen, Tongfeng Zhao

**Affiliations:** 1 Departments of Endocrinology, the Sixth Affiliated Hospital, Sun Yat-Sen University, Guangzhou, 510655 China; 2 Departments of Nephrology, the Sixth Affiliated Hospital, Sun Yat-Sen University, Guangzhou, 510655 China

**Keywords:** Ubiquitylation, Cardiovascular diseases

## Abstract

Extracellular acidosis-induced mitochondrial damage of cardiomyocytes leads to cardiac dysfunction, but no detailed mechanism or efficient therapeutic target has been reported. Here we found that the protein levels of MIC60 were decreased in H9C2 cells and heart tissues in extracellular acidosis, which caused mitochondrial damage and cardiac dysfunction. Overexpression of MIC60 maintains H9C2 cells viability, increases ATP production and mitochondrial membrane potential, mitigates the disruptions of mitochondrial structure and cardiac injury. Mechanistically, extracellular acidosis excessively promoted MIC60 ubiquitin-dependent degradation. TRAP1 mitigated acidosis-induced mitochondrial impairments and cardiac injury by directly interacting with MIC60 to decrease its ubiquitin-dependent degradation in extracellular acidosis.

## Introduction

Body fluid comprises extracellular fluid and intracellular fluid, wherein the extracellular fluid mainly comprises endovascular fluid (in the form of plasma) flowing within blood vessels and tissue interstitial fluid [1]. Both the endovascular fluid and interstitial fluid constitute the liquid environment of cells in the body, namely the internal environment [2]. Maintaining the stability of the internal environment is of great significance in ensuring the normal physiological function of the body. Many factors can affect the stability of the internal environment, in which the acid-base balance of extracellular fluid and pH regulation play important roles [3]. In the clinic, the abnormal acid-base balance of extracellular fluid can be induced by many diseases. In acid-base disorders, extracellular acidosis is a common pathophysiological change, which can occur in the whole body or local microenvironment of tissue and organ [4]. Metabolic acidosis is the most common cause of systemic extracellular acidosis, which is often caused by serious dysfunction of organs (such as heart, liver, kidney, and lung) and diabetes mellitus with severe hyperglycemia [5]. Extracellular acidosis occurring in the local microenvironment is often caused by aging, inflammation, infection, tumor, and diabetic microvascular disease [6]. In these cases, due to much less capacity of pH buffers than that in blood, the pH of interstitial fluids would be changeable out of the normal range, while the arterial blood pH stays within the normal range [7, 8]. It has been found that severe acidosis of the local microenvironment can reduce the pH of interstitial fluid to 6.5–6.0 [9].

The heart is an important circulatory dynamic organ and is also sensitive to pH changes [10, 11]. Extracellular acidosis leads to cardiac injury and dysfunction, which is associated with poor clinical outcome [12, 13]. Clarifying the mechanisms and finding a potential therapeutic target is meaningful. Studies reported that extracellular acidosis induced a negative inotropic effect in primary cardiomyocytes via changing the sensitivity to calcium andβ-adrenergic [14]. Furthermore, extracellular acidosis damaged heart tissue structure and induced left ventricular dysfunction in rats [15]. In our previous study, we found that extracellular acidosis damaged mitochondria and activated mitochondrial apoptosis pathway in H9C2 cells (rat myocardial cell line) in extracellular acidosis [16], which suggested that mitochondria might play an important role in extracellular acidosis-induced cardiac dysfunction.

Mitochondria is very crucial for cardiomyocytes as an energy supplier [17]. Extracellular acidosis severely impaired mitochondrial function and decreased the ATP levels of H9C2 cells in our previous study [16]. A similar study also reported that extracellular acidosis decreased mitochondrial membrane potential and induced mitochondrial permeability transition pore (mPTP) associated cell death in HEK293 cells [4]. Noteworthy, ischemia activated mitochondrial apoptosis pathway of endothelial cells might highly depend on cellular acidosis but not hypoxia, suggesting the unique role of acidosis in ischemic disease [18, 19]. These results identified that mitochondrial injury might play an important role in extracellular acidosis-induced cardiac dysfunction.

Mitochondria are comprised of outer membrane, inner membrane, and cristae [20]. Mitochondrial cristae are the main sites of ATP production and maintaining its normal structure is very crucial for mitochondrial function [21]. Abnormal mitochondrial cristae structure played a significant role in the progressions of several diseases [22, 23]. In our previous study, we found that extracellular acidosis disrupted normal mitochondrial structure, including cristae, which suggested mitochondrial structure impairment might involve extracellular acidosis-induced mitochondrial dysfunction. More importantly, we found that tumor necrosis factor receptor-associated protein 1 (TRAP1) partially reversed mitochondrial structure impairment in extracellular acidosis [16], which suggested that TRAP1 might be a potential therapeutic target for this process.

TRAP1 belonged to the heat shock protein 90 family and was mainly located in mitochondria [24]. Previous studies reported that TRAP1 mitigated mitochondrial oxidative stress damage, maintained mitochondrial integrity, and regulated oxidative phosphorylation under multiple conditions, including ischemia, hypoxia, and endoplasmic reticulum stress [25–27]. These studies suggested that TRAP1 protected mitochondrial function in various diseases. In our study, we found TRAP1 partially reversed mitochondrial cristae structure in extracellular acidosis, suggesting that TRAP1 might participate in mitochondrial ultrastructure regulation in extracellular acidosis. But the detailed mechanisms were still unknown.

In order to clarify how TRAP1 regulated mitochondrial structure, we used immunoprecipitations (IP) combined with liquid chromatography-tandem mass spectrometry (LC-MS/MS), and found a core component of mitochondrial cristae, mitochondrial contact site and cristae organizing system subunit 60 (MIC60), which is interacted with TRAP1. MIC60 is very important for normal mitochondrial cristae structure [28]. Previous studies reported that MIC60 was altered and misfolded under several different pathological conditions [29, 30]. To maintain homeostasis in cells, misfolded proteins may be refolded to normal structure with the help of molecular chaperones, or degraded through a ubiquitin-proteasome system or lysosome [31]. TRAP1 was reported to regulate mitochondrial proteins quality control and ubiquitination [32].

In our present study, we confirmed that TRAP1 interacted with MIC60. Moreover, extracellular acidosis promoted MIC60 ubiquitin-dependent degradation to damage the normal mitochondrial structure of rat cardiomyocytes. TRAP1 decreased MIC60 ubiquitination to maintain normal mitochondrial function and structure and cardiac function.

## Results

### TRAP1 directly interacts with MIC60

**Fig. 1:**
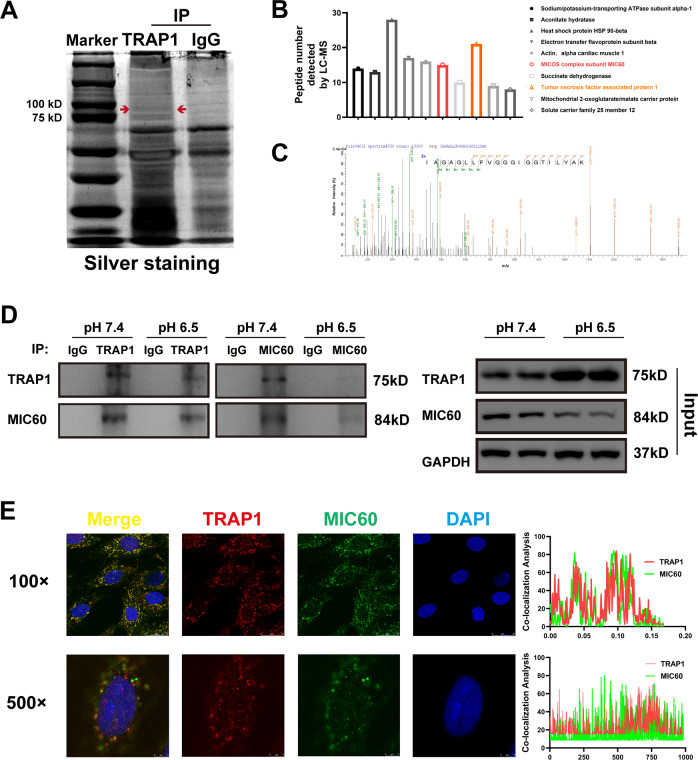
TRAP1 directly interacted with MIC60 in H9C2 cells. **A** Silver staining of TRAP1 immunoprecipitates. **B** LC-MS/MS of TRAP1 immunoprecipitates. **C** Representative fragmentation spectrum of the identified MIC60 peptides. **D** Immunoblotting analysis of TRAP1 and MIC60 expression in a co-IP assay performed in H9C2 cells with anti-TRAP1 or anti-MIC60 Magnetic beads, respectively. **E** Detection of the colocalization of MIC60 (green) and TRAP1 (red) using confocal microscopy in H9C2 cells. Nuclei were stained using DAPI (blue), (Scale bars, 25 um). Data were the means ± SD from three independent experiments.

To identify potential proteins that TRAP1 interacted with and regulated to protect mitochondrial function and structure, we performed TRAP1 immunoprecipitates, resolved IP-sample in SDS-PAGE, and performed silver staining (Fig. 1A). Then we conduct LC-MS of the marker gel and found MIC60, an important component forming mitochondrial cristae (Fig. 1B, C). Further, we confirmed the interaction of TRAP1 and MIC60 via CO-IP assay (Figs. 1D and S2A) and immunofluorescence colocalization (Fig. 1E). To identify the specificity of the interaction between TRAP1 and MIC60, we detected whether other mitochondrial proteins could interact with TRAP1, including Prohibitin, VDAC, and TOM20. And we did not observe interactions of TRAP1 with Prohibitin, VDAC, or TOM20 (Fig. S2A).

### Decreasing MIC60 protein levels is crucial in extracellular acidosis-induced mitochondrial damage

**Fig. 2:**
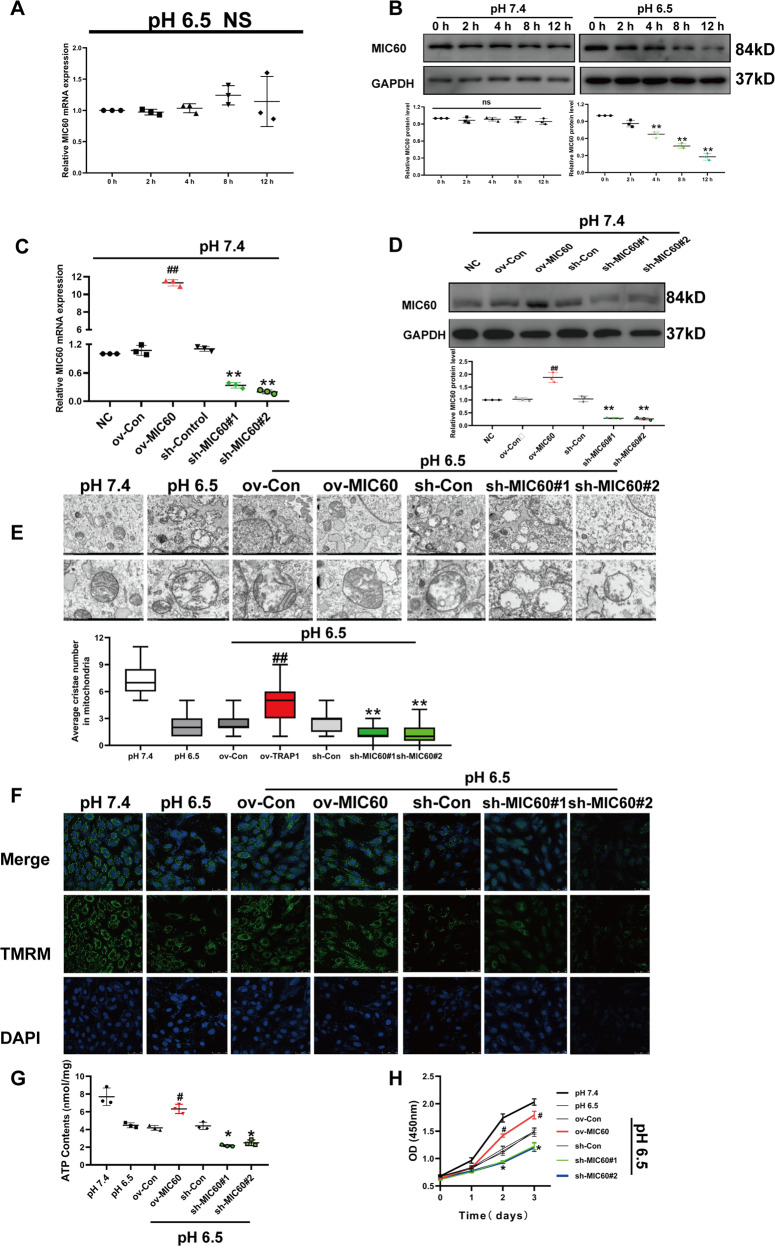
Decreasing MIC60 protein levels were a key process in extracellular acidosis-induced mitochondrial cristae and function injury. **A**, **B** RT-qPCR and western blot were used to determine the mRNA and protein levels of MIC60 in extracellular acidosis (pH 6.5). **C**, **D** RT-qPCR and western blot were used to determine the transfection efficiency of MIC60 in H9C2 cells. **E** Transmission electron microscopy was used to detect the ultrastructure of mitochondria of H9C2 cells. Twenty-one images were obtained from three independent experiments for each group. The average cristae number in one mitochondrion (*n* = 30 mitochondria for each group randomly selected from 21 pictures) of each group was quantified. **F** Methyl tetramethylrhodamine staining was used to detect mitochondrial membrane potential of H9C2 cells. **G** ATP assay was used to detect ATP levels of H9C2 cells. **H** CCK8 assay was used to detect the cell viability of H9C2 cells. Data were the means ± SD from three independent experiments. Group comparisons were performed by one-way analysis of variance followed by Tukey’s post hoc test. NS: no statistically significant difference between groups. **P < 0.01 vs. ov-Con group. ^##^*P* < 0.01 vs. sh-Con group.

Even though TRAP1 directly interacted with MIC60, we did not know whether TRAP1 regulated MIC60 to protect the mitochondrial structure in extracellular acidosis. So we should first identify whether MIC60 plays an important role in extracellular acidosis-induced mitochondrial damage. We observed no significant changes in MIC60 mRNA levels (Fig. 2A), but an obvious decrease in protein levels in extracellular acidosis (Fig. 2B). To confirm the decreasing of MIC60 protein was specific but not due to the reduced mitochondrial number, we detect the protein levels of other mitochondrial proteins, including Prohibitin, VDAC, and Tom20. No significant decrease of these proteins was observed (Fig. S2B). Next, we constructed MIC60 overexpression H9C2 cells and MIC60 silencing H9C2 cells (Fig. 2C, D) and found that MIC60 overexpression could decrease mitochondrial structure injury (Fig. 2E), improve mitochondrial membrane potential (Fig. 2F), ATP contents (Fig. 2G), and cell proliferation (Fig. 2H). These results confirmed that decreasing MIC60 protein levels played an important role in extracellular acidosis-induced mitochondrial injury and restoring MIC60 protein levels could decrease the injury.

### TRAP1 restores MIC60 protein levels in extracellular acidosis via regulating ubiquitin-dependent degradation of MIC60

**Fig. 3:**
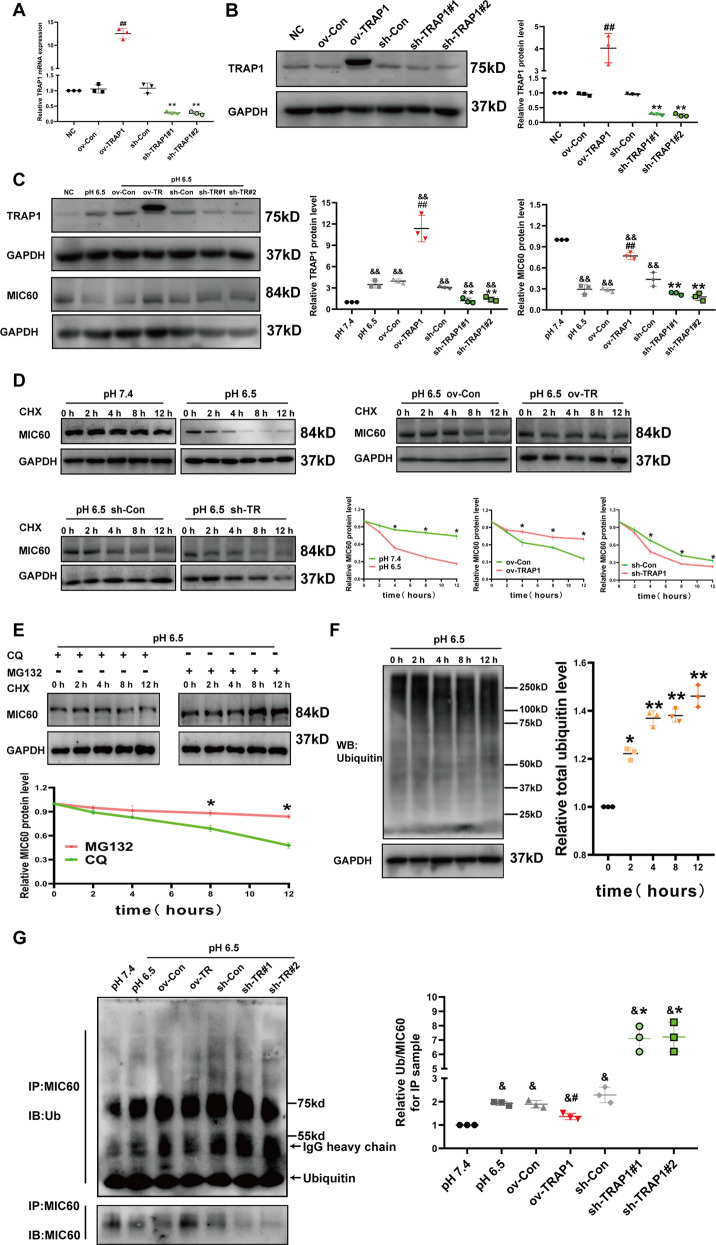
TRAP1 increased MIC60 protein levels of H9C2 cells via alleviating MIC60 ubiquitin-dependent degradation in extracellular acidosis. **A**, **B** RT-qPCR and western blot were used to determine the transfection efficiency of TRAP1 in H9C2 cells. **C** Western blot was used to detect TRAP1 or MIC60 protein levels of H9C2 cells. **D**, **E** Western blot was used to detect the degradation of MIC60 protein. **F** Western blot was used to detect whole cell lysate ubiquitination. **G** Western blot was used to detect MIC60 protein ubiquitination. CHX (cycloheximide, 20 uM) was used to inhibit protein synthesis. MG132 (10 uM) was used to inhibit proteasome. CQ (Chloroquine phosphate, 10 uM) was used to inhibit lysosomes. Data were the means ± SD from three independent experiments. Group comparisons were performed by one-way analysis of variance followed by Tukey’s post hoc test. ^&&^*P* < 0.01 vs. pH 7.4 group. ^##^*P* < 0.01 vs. ov-Con group. *P < 0.05 vs. 0 h, CQ or Con group. **P < 0.01 vs. sh-Con or 0 h group.

After identifying the crucial role of MIC60 in extracellular acidosis-induced mitochondrial injury, we wanted to know whether TRAP1 played a protective role via regulating MIC60. We conducted TRAP1 overexpression of H9C2 cells and TRAP1 silencing H9C2 cells (Fig. 3A, B) and found that TRAP1 could restore MIC60 protein levels in extracellular acidosis (Fig. 3C). Because we proved that MIC60 protein levels, but not mRNA levels, was obviously decreased in extracellular acidosis (Fig. 2A, B), we speculated that extracellular acidosis promoted MIC60 protein degradation to decrease its protein levels and TRAP1 decreased the degradation. To prove this speculation, we used cycloheximide (CHX, 20 uM) to inhibit new protein synthesis, and then observed extracellular acidosis truly promoted MIC60 protein degradation and TRAP1 could partly decrease this process (Fig. 3D). Furthermore, proteasome inhibitor MG132 (10uM) could significantly inhibit acidosis-induced MIC60 degradation, but not Lysosomal inhibitor CQ (10 uM) (Fig. 3E). This suggested that extracellular acidosis might promote MIC60 protein degradation through a ubiquitin-proteasome system (UPS) dependent way. So we detected the ubiquitin levels of whole cell lysate and found that extracellular acidosis promoted ubiquitin levels of total proteins (Fig. 3F). Similarly, ubiquitin levels of MIC60 was increased in extracellular acidosis, and TRAP1 could decrease MIC60 ubiquitination (Figs. 3G and S2C). But we did not observe TRAP1 decreased total protein ubiquitination in extracellular acidosis (data not shown).

### TRAP1 protects mitochondria by increasing MIC60 protein levels in extracellular acidosis

**Fig. 4:**
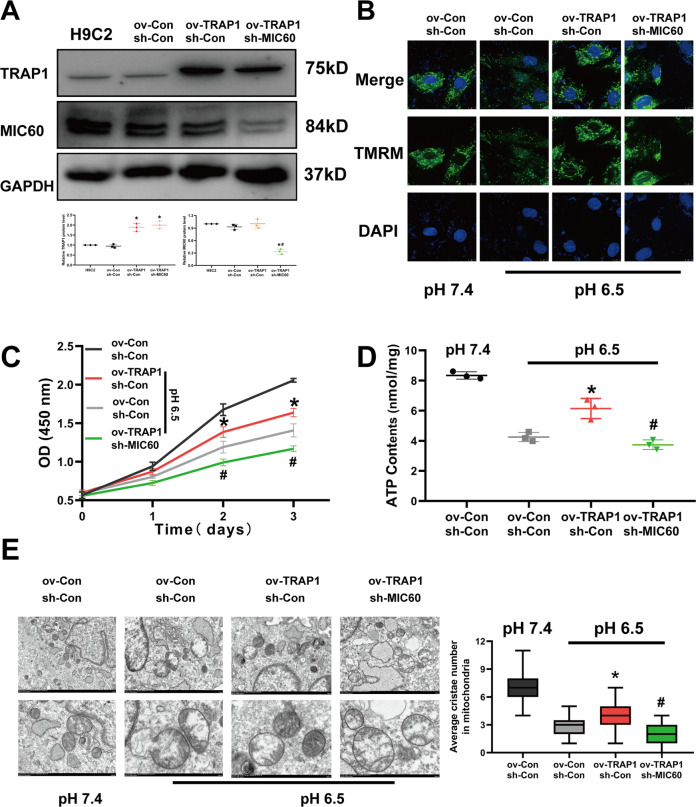
TRAP1 protected mitochondrial cristae and function by increasing MIC60 protein levels in extracellular acidosis. **A** Western blot was used to determine the transfection efficiency of TRAP1 and MIC60 in H9C2 cells. **B** Methyl tetramethylrhodamine staining was used to detect mitochondrial membrane potential of H9C2 cells. **C** CCK8 assay was used to detect the cell viability of H9C2 cells. **D** ATP assay was used to detect ATP levels of H9C2 cells. **E** Transmission electron microscopy was used to detect the ultrastructure of mitochondria of H9C2 cells. Twenty-one images were obtained from three independent experiments for each group. The average cristae number in one mitochondrion (*n* = 30 mitochondria for each group randomly selected from 21 pictures) of each group was quantified. Data were the means ± SD from three independent experiments. Group comparisons were performed by one-way analysis of variance followed by Tukey’s post hoc test. *P < 0.05 vs. ov-Con/sh-Con group. ^#^*P* < 0.05 vs. ov-TRAP1/sh-Con group.

We further detected whether TRAP1 protected mitochondria by increasing MIC60 protein levels in extracellular acidosis. We conducted TRAP1 overexpression and MIC60 silencing H9C2 cells (Fig. 4A). We found that the protective effects of TRAP1 overexpression on mitochondrial membrane potential (Fig. 4B), cell viability (Fig. 4C), ATP levels of H9C2 cells (Fig. 4D), and mitochondrial structure (Fig. 4E) were abolished when MIC60 was knocked down in the meantime. These results suggested the protective effects of TRAP1 on mitochondria were highly dependent on MIC60.

### TRAP1 interacts with MIC60 and regulates MIC60 ubiquitination to increase MIC60 protein levels in rats’ heart tissue in extracellular acidosis

**Fig. 5:**
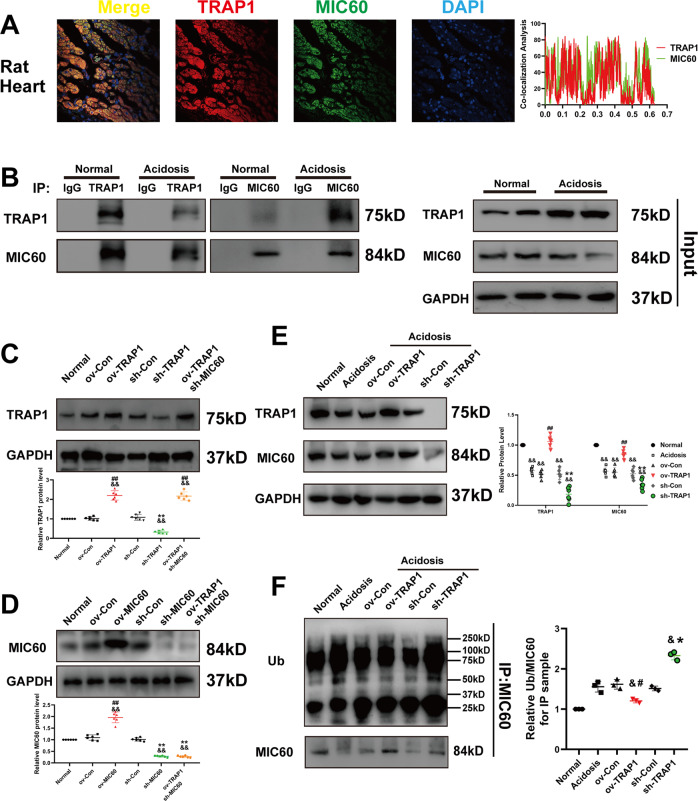
TRAP1 interacted with MIC60 and decreased MIC60 ubiquitination to increase MIC60 protein levels in rats’ heart tissue in extracellular acidosis. **A** Detection of the colocalization of MIC60 (green) and TRAP1 (red) using confocal microscopy in rat’s heart tissue. **B** Immunoblotting analysis of TRAP1 and MIC60 expression in a co-IP assay performed in protein lysate of rat’s heart tissue with anti-TRAP1 or anti-MIC60 Magnetic beads, respectively. **C**–**D** Western blot was used to determine the transfection efficiency of TRAP1 (**C**) and MIC60 (**D**) in rats’ heart tissue. **E** Western blotting was used to detect MIC60 protein levels regulated by TRAP1. **F** Western blot was used to detect MIC60 protein ubiquitination. Data were the means ± SD from three independent experiments. Group comparisons were performed by one-way analysis of variance followed by Tukey’s post hoc test. ^&^*P* < 0.05 vs. Normal group. ^#^*P* < 0.05 vs. ov-Con group. ^##^*P* < 0.01 vs. ov-Con group. *P < 0.05 vs. sh-Con group. **P < 0.01 vs. sh-Con group.

We conducted in vivo experiments to further confirm our results. We found that TRAP1 co-localized with MIC60 in rat’s heart tissue (Fig. 5A) and they directly interacted with each other (Fig. 5B). Next, considering the protein length of TRAP1 or MIC60 was out of rAAV loading capacity, we constructed lentiviral vectors to perform transgenic rat in cardiac tissue, and the transfection efficiencies of TRAP1 or MIC60 were confirmed by western blot and immunohistochemical staining (Figs. 5C, D, and S1C). We found that TRAP1 in vivo increased MIC60 protein levels (Fig. 5E) and decreased MIC60 ubiquitination (Fig. 5F) in extracellular acidosis, which is consistent with our in vitro results.

### TRAP1 protects cardiac function and mitochondrial structure of rats’ heart by increasing MIC60 protein levels in extracellular acidosis

**Fig. 6:**
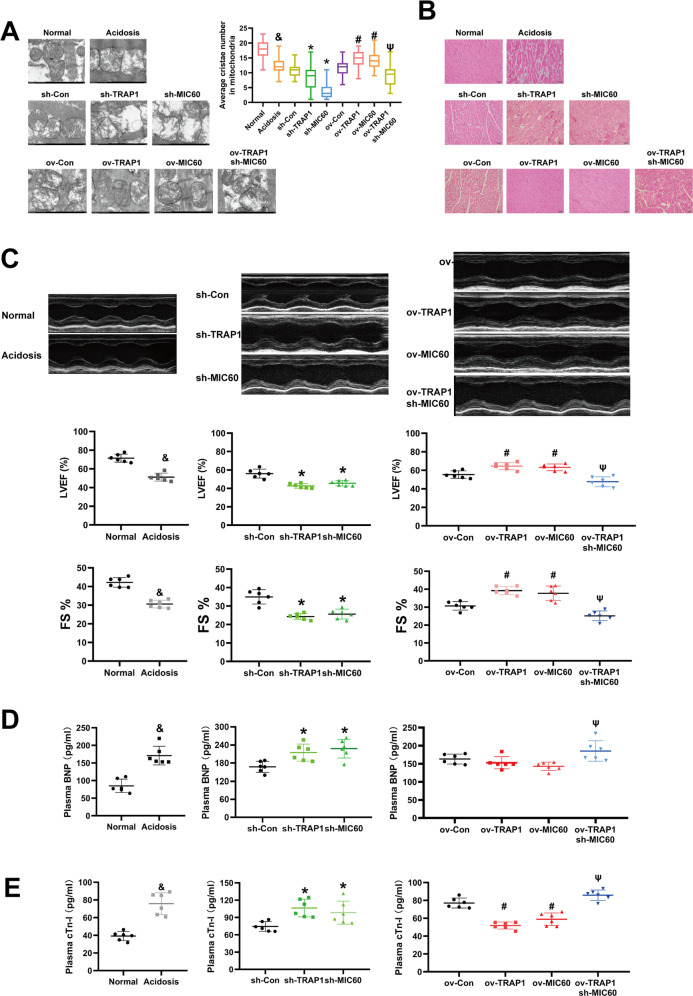
TRAP1 protected cardiac function and mitochondrial cristae of rats’ heart by increasing MIC60 protein levels in extracellular acidosis. **A** Transmission electron microscopy was used to detect the ultrastructure of mitochondria of rats’ heart tissue. Twenty-five images were obtained from five rats for each group. The average cristae number in one mitochondrion (*n* = 30 mitochondria for each group randomly selected from 25 pictures) of each group was quantified. **B** HE staining was used to detect tissue injury of rats’ heart tissue. **C** Echocardiography was used to detect cardiac functions of rats including LVEF. **D**–**E** Elisa assay was used to detect serum BNP (**D**) and cTnI (**E**). Data were the means ± SD from six rats. Group comparisons were performed by one-way analysis of variance followed by Tukey’s post hoc test. ^&^*P* < 0.05 vs. Normal group. *P < 0.05 vs. sh-Con group. ^#^*P* < 0.05 vs. ov-Con group. ^ψ^*P* < 0.05 vs. ov-TRAP1 group or ov-MIC60 group.

Finally, we detected whether the protective effects of TRAP1 and MIC60 on mitochondria could improve cardiac function and decrease histological injury. We found that either TRAP1 overexpression or MIC60 overexpression could obviously decrease mitochondrial (Fig. 6A) and histological injury (Fig. 6B) of heart tissue. Furthermore, either TRAP1 overexpression or MIC60 overexpression increased LVEF and FS (Fig. 6C), decrease serum BNP (Fig. 6D), and cTnI (Fig. 6E).

## Discussion

In the current study, we identified TRAP1 directly interacted with MIC60 and decreased MIC60 ubiquitination to restore MIC60 protein levels in cardiomyocytes, which significantly mitigated mitochondrial and cardiac injury in extracellular acidosis.

It has been proposed long before that extracellular acidosis led to left ventricular dysfunction and series theories were put forward according to the observations [11, 15]. In recent years, researchers reported that extracellular acidosis severely impaired the mitochondrial function of HEK293 cells [4]. Considering the important role of mitochondria in cardiomyocytes, it was crucial to identify whether extracellular acidosis impaired mitochondria of cardiomyocytes. At first, we identified extracellular acidosis (pH 6.5) severely damaged mitochondrial function and structure of H9C2 cells [16]. Mitochondrial cristae, on which mitochondrial respiratory chain-associated proteins were bound, is an important component of the mitochondrial inner membrane and very crucial for mitochondrial oxidative phosphorylation [33, 34]. Previous studies reported that mitochondrial cristae damage led to severe mitochondrial dysfunction and activated mitochondrial apoptosis pathway [35, 36]. Our results also demonstrated that mitochondrial structure damage including cristae might be a key process in extracellular acidosis-induced mitochondrial injury. Moreover, we found TRAP1 restored mitochondrial structure and mitochondrial function in extracellular acidosis. To identify how TRAP1 protected mitochondrial structure, we performed CO-IP and LC-MS and identified that TRAP1 directly interacted with MIC60 (Fig. 1).

MIC60 is an important component of mitochondrial cristae [37]. Previous studies reported that the decreasing MIC60 protein levels played a significant role in mitochondrial cristae disruption [38, 39]. Here we found that extracellular acidosis significantly decreased MIC60 protein levels and exogenous overexpression of MIC60 restored mitochondrial structure (Fig. 2F). This suggested that decreasing MIC60 protein levels plays an important role in extracellular acidosis-induced mitochondrial damage. Furthermore, we did not observe a significant change in MIC60 mRNA levels (Fig. 2A). We speculated that extracellular acidosis might promote MIC60 protein degradation after translation, the following assay confirmed our speculations (Fig. 3D). Our study showed that, in extracellular acidosis, MIC60 protein was degraded by a ubiquitin-proteasome system (UPS) dependent way (Fig. 3E and F), and TRAP1 could increase MIC60 protein levels by inhibiting MIC60 ubiquitination (Fig. 3C, G). UPS was considered to be involved in mitochondrial proteins homeostasis, including mitochondrial inner [40] or outer membrane proteins [41, 42]. Through altering functional proteins’ stasis in physiological or pathological conditions, UPS could regulate mitochondrial functions including autophagy [43] and energy metabolism [44].

In addition, heat shock protein families also participated in regulating proteins homeostasis in coordinating with UPS both in mitochondria [45] and cytoplasm [46, 47]. Most mitochondrial proteins are transcribed in the nucleus, translated into the cytoplasm, and then imported into mitochondria. TRAP1 belongs to the heat shock protein 90 (HSP90) family, mainly located in mitochondria [48]. A previous study reported that TRAP1 regulated the ubiquitination and mitochondrial importing of mitochondrial inner proteins such as Sorcin and F1ATPase in cytosol coordinating with the proteasome regulator particles TBP7 to regulate multiple cellular functions. [49, 50]. Here we found that TRAP1 decreased MIC60 ubiquitination to restore normal mitochondrial formation in extracellular acidosis (Fig. 4E). According to the researches above, it is highly suggested that HSP families coordinating with UPS played a significant role in regulating proteins homeostasis. But we have not demonstrated whether this process happened in cytosol or mitochondria. More extensive and detailed researches will be needed to investigate in the future.

Finally, we further studied whether TRAP1-MIC60 regulation could improve rats’ cardiac function in extracellular acidosis. Because the protein length of TRAP1 or MIC60 was out of rAAV loading capacity, we chose lentiviral vectors (100 ul vectors (2 × 10^8^ TU/ml) per rat) in situ injection to construct transgenic rats in hearts (Fig. S1A) [51–53]. In cardiac tissue, we identified that TRAP1 could directly interact with MIC60 (Fig. 5A), and increase MIC60 protein levels by decreasing its ubiquitination in acidosis rats (Fig. 5E, F). TRAP1 also partly restored normal mitochondrial structure in heart tissue and this effect was dependent on MIC60 (Fig. 6A, B). More importantly, TRAP1 increased LVEF of rats’ heart (Fig. 6C) and deceased serum BNP/cTnI levels (Fig. 6D, E) in acidosis rats, which suggested that the TRAP1-MIC60 pathway might be an effective therapeutic target to decrease cardiac injury and improve cardiac function for patients with acidosis.

In our present study, we used lentiviral vectors to construct transgenic rats (Fig. S1A). Lentiviral vectors have unique advantages in gene delivery, including high transfection efficiency to both replicating and non-replicating cells, large loading capacity, and low immunogenicity [54]. Therefore lentiviral vectors were considered to be an ideal vector for gene therapy, including in cardiovascular disease [55]. However, lentiviral vectors stably integrated into the genome of target cells, which brought concerns about its potential carcinogenicity [56]. With the developments of constructing technology of lentiviral vectors, third-generation lentiviral vectors were identified sufficient safety and effectiveness [57, 58], but wide clinical applications of lentiviral vectors still need a long way to go.

## Methods

### Cell culture

Rat myocardial cell line (H9C2) was purchased from the Cell Bank of the Chinese Academy of Sciences (GNR 5). Cells were cultured in Dulbecco’s modified Eagle’s medium (DMEM, 11965175, Gibco; Thermo Fisher Scientific, Inc.) supplemented with 1.5 g NaHCO3/L, 10% fetal bovine serum (10099141 C, Gibco, Thermo Fisher Scientific, Inc.), and 1% penicillin/streptomycin (cat. no. 516106, Sigma-Aldrich) at 37 °C in 5% CO_2_. For in vitro experiment, the pH value of the cell culture medium was adjusted using 2-(*N*-morpholino) ethanesulfonic acid [59] (MES, 30 mM, M3671, Sigma-Aldrich).

### Protein lysate

H9C2 cells or rats’ heart tissue were lysed in cell lysis buffer for Western and IP (P0013, Beyotime, China) supplemented with protease inhibitor cocktail (78430, Thermo Fisher Scientific, Inc.) for 30 min on ice. Then heart tissue were grinded (30 HZ, 4 °C) for 15 min in a milling machine (MM400, Retsch, Germany). After centrifuging at 4 °C at 12,000 × *g* for 20 min, the supernatants were collected. Protein concentrations were measured by BCA Assay Kit (23225, Thermo Fisher Scientific, Inc.).

### Immunoprecipitation assay

Protein A/G magnetic beads (HY-K0202, MedChemExpress, USA) were incubated with indicated antibody (TRAP1, sc-13557, Santa Cruz; MIC60, ab110329, Abcam) at 4 °C for 2 h. After washing beads with PBST (PBS and 0.5% Triton X-100) five times, protein lysate containing 500 μg total protein were incubated with beads at 4 °C for 6 h. Beads were washed with PBST five times after incubation, then boiled at 95 °C for 10 min with 1× SDS-PAGE loading buffer. Proteins were detected by silver staining (P0017S, Beyotime, China), liquid chromatography-tandem mass spectrometry (LC-MS), or Western Blot after being separated in 8% SDS-poly-acrylamide (SDS-PAGE) gel. Protein lysate containing 25 μg total protein were detected as input.

### Liquid chromatography-tandem mass spectrometry (LC-MS/MS)

LC-MS assay were conducted by Fitgene Biotechnology Co. (Guangzhou, China). Briefly, After separating in SDS-PAGE gel and performing silver staining, interest blots were collected and digested. Residing peptides were extracted and dissolved in 2% acetonitrile and 0.1% formic acid. Liquid chromatography assay was performed using Acclaim PepMap RSLC C18 (Thermo, 160454) and Acclaim PepMap 75 um × 150 mm (Thermo, 160321). Separated peptides were analyzed with the mass spectrometer (Thermo Scientific Q Exactive). MS data were searched on Sorcerer2-SEQUEST using the reviewed Swiss-Prot database.

### Immunofluorescence colocalization

For in vitro experiment, cells were washed with cold PBS three times, fixed with 4% paraformaldehyde at 37 °C for 15 min, and permeabilized with 0.1% Triton X-100 at room temperature for 15 min. Then, cells were blocked with 5% BSA for 30 min before being incubated with indicated primary antibodies (TRAP1, NBP2-47597, Novus; MIC60, ab110329, Abcam) and secondary antibody (ab150113 and ab150078, Abcam) at 4 °C for 8 h, respectively.

For in vivo experiments, paraffin slides of rats’ heart tissue (after dewaxing and antigen retrieval) were permeabilized with 0.1% Triton X-100 at room temperature for 20 min and blocked with 5% BSA for 30 min. Antibodies incubation were the same as above.

Fluorescence was observed using a laser scanning confocal microscope (Leica TCS SP8). Colocalization analysis was performed using ImageJ (V2.1.4.8; National Institute of Health).

### RNA isolation, reverse transcription (RT), and quantitative real-time qPCR (RT-qPCR)

Total cellular RNA was extracted using TRIzol reagent (15596018, Invitrogen) and total RNA density was detected using Nanodrop (Thermo). Then RT-PCR were conducted using a kit (RR036A, Takara). Real-time quantitative PCR (RT-qPCR) was carried out using another kit (RR420A, Takara). And the primer pairs used were listed:

1. TCAGTCAGAGGCTAAGGTGGTGTC and TGGAGAGTGTGCCAGCTAGGTC for rat MIC60;
2. ACGGCAAGTTCAACGGCACAG and CGACATACTCAGCACCAGCATCAC for rat GAPDH;
3. AGGTGTGGTGGACAGTGAGGAC and GCATTCGGCTGGCGTAGTCTG for rat TRAP1.

### Western blot and protein half-life analysis

Protein lysate containing 20 μg total proteins were boiled at 95 °C for 10 min with 5× SDS-PAGE loading buffer, then separated in 6 or 8% sodium dodecyl sulfate-polyacrylamide gel electrophoresis (SDS-PAGE). After transferring proteins to PVDF membrane (IPVH00010; Millipore, USA) and blocking in 5% non-fat milk (P0216; Beyotime, China) for 1 h at room temperature, membrane was incubated with primary antibodies (TRAP1, NBP2-47597, Novus; MIC60, 10179-1-AP, Proteintech; Ubiquitin, 3936 S, CST) at 4 °C overnight and incubated with secondary antibodies for 1 h at room temperature. Bands were visualized by chemiluminescence with Immobilon Western Chemiluminescent HRP Substrate (WBKLS; Millipore) on ChemiDoc Imaging System (Bio-Rad, CA, USA). Semi-quantifications of bands were performed using ImageJ (V2.1.4.8; National Institute of Health).

For the protein half-life analysis, cells were treated with cycloheximide (CHX, 20 uM, HY-12320, MedChemExpress), MG132(10 uM, HY-13259, MedChemExpress), and Chloroquine phosphate (CQ, 10 uM, HY-17589A, MedChemExpress) for different times.

### Cell transfection

Lentiviral particles (GeneChem, Shanghai, China) were used for overexpression or silencing TRAP1 and MIC60. Targeting sequence of small interfering RNA (siRNA) against rat MIC60 was 5′-AAGGTGGTGTCTCAATATCAT-3′ (#1) and 5′-TTGGAGCACCATAGAAGTGAA-3′(#2). The targeting sequence of small interfering RNA (siRNA) against rat TRAP1 was 5′-TACGATAAGCCTCGCTTCATT-3′(#1) and 5′-CAGGCACACACTAATAAAGAA-3′(#2). Multiplicity of infection (MOI) for H9C2 cells was 10. At 72 h post-infection, cells were selected by puromycin to obtain H9C2 cells stably overexpression TRAP1 or TRAP1 silencing. The transfection efficiency was identified by PCR and western blot.

### Mitochondrial membrane potential (MMP) assay

Tetramethylrhodamine, methyl ester (TMRM; cat. no. I34361; Thermo Fisher Scientific) was used for detecting MMP according to the manufacturer’s instructions. Briefly, live cells were cultured with 50 nmol/l TMRM in serum-free medium at 37 °C for 30 min, washed with PBS three times, and then observed under a confocal microscope (Leica TCS SP8).

### Cell viability

Cell Counting kit-8 (CK04; Dojindo) was used for detecting cell viability according to the instruction. Briefly, cells were seeded into 96-well plates at a density of 2000 cells per well in complete culture medium. About 10 μl testing solution was added to each well after processing, incubating for an additional 1.5 h. Optical density values were measured at a 450-nm wavelength on a microplate reader (Multiskan^TM^ FC; Thermo Fisher Scientific, Inc.).

### ATP assay

ATP levels were detected using an Enhanced ATP Assay kit (S0027, Beyotime) according to the instructions. Briefly, cells in different groups were added with 100 μl lysis buffer provided with the kit and cell lysate were centrifuged at 12,000 × *g* at 4 °C for 20 min.About 10 μl supernatant was mixed with 100 μl of ATP detection solution. Then luminescence intensity was measured using a luminometer (Varioskan Flash; Thermo Fisher Scientific). The luminescence intensities of ATP standards were determined in a similar manner. The ATP concentration was calculated according to an ATP-standard curve and normalized to protein concentrations of the supernatants.

### Transmission electron microscopy

The mitochondrial ultrastructure were observed using a transmission electron microscope (TEM, Hitachi HT7700, TO, Japan). H9C2 cells or fresh myocardium sections were collected and fixed in 4% glutaraldehyde for 1 h at room temperature and at 4 ˚C overnight. The samples were dehydrated through a graded ethanol series, then incubated in 100% ethanol and propylene oxide as well as two exchanges of pure propylene oxide. Samples were embedded in epoxy resin and polymerized at 60 ˚C for 48 h. Specimens were cut into 70–80 nm ultra-thin sections, then mounted on 300-mesh copper grids. Sections were stained with uranyl acetate and leas citrate, then subjected to observation.

### Animal study

Male Sprague-Dawley rats (8 weeks) were purchased from Charles River, China (Beijing, under the license of Charles River, USA). Animal studies were performed in accordance with EU Directive 2010/63EU and Recommendation 2007/526/EC regarding the protection of animals used for experimental and other scientific purposes. The protocol of present study was approved by the ethical committee of Sun Yat-Sen University (IACUC-2020041304). For study design, the researchers were not blinded to the experiments. Fifty-four rats were randomly divided into nine groups (six for each group), among which none was excluded: (I) normal rats; (II) acidosis rats; (III) acidosis rats transfected with lentiviral over-control; (IV) acidosis rats transfected with lentiviral TRAP1; (V) acidosis rats transfected with lentiviral MIC60; (VI) acidosis rats transfected with lentiviral scramble shRNA; (VII) acidosis rats transfected with lentiviral TRAP1 shRNA; (VIII) acidosis rats transfected with lentiviral MIC60 shRNA; and (IX) acidosis rats transfected with lentiviral TRAP1 and lentiviral MIC60 shRNA.

Transgenic rats overexpression or silencing TRAP1 and MIC60 in heart tissue were obtained through in situ injection. Briefly, 100 ul lentiviral vectors (2 × 10^8^ TU/ml) were injected at 4–5 points on cardiac impulsing area using a 28-gauge needle (about 20 μl per site) [60] (Fig. S1A). Transfection efficiency was confirmed by western blot and immunohistochemical staining.

Acidosis rats were conducted as described before [15] at 7 days after lentiviral vectors injection. Acidosis rats received orally 0.28 M ammonium chloride in drinking water for 2 weeks ad libitum. Daily acid load administered was 11 mM per rat approximately. Arterial blood pH was detected using a blood gas analyzer (ABL80FLEX, Radiometer Medica) at the end of the experiments.

### Echocardiography

Rats were anesthetized with 2% isoflurane in room air. Then, a Vevo 2100 system (VisualSonics, USA) was used for transthoracic echocardiography analysis (30 MHz sectorial probe). Systolic and diastolic parameters were measured using the two-dimensional parasternal long-axis view at the levels of the papillary muscle.

### Histological analysis

Formalin-fixed, paraffin-embedded tissue were cut into 4 um thick sections. For immunohistochemical staining and immunofluorescence, sections were dewaxed with xylene and rehydrated by a graded series of alcohols, followed by antigen retrieval and blocked with 5% BSA for 30 min and permeabilized with 0.10% Triton X-100 at room temperature for 20 min. Then slides were incubated with 1.5% hydrogen peroxide solution for 5 min to block endogenous peroxidase. After incubating with primary and HRP conjugated secondary antibodies respectively, sections were staining with DAB solution (ZLI-9018, ZsBio, China). Haematoxylin and eosin staining was performed using a staining kit (C0105S, Beyotime). Images were acquired with an upright microscope (BX57, Olympus, Japan).

### Statistical analysis

Data represent the mean ± SD from three independent experiments and were analyzed using SPSS 25.0 (IBM Corp.). One-way analysis of variance followed by Tukey’s post hoc test was carried out to measure differences between groups. *P* < 0.05 was considered to indicate a statistically significant difference.

## Supplementary information

Supplemental figure legends

Figure S1

Figure S2

Dataset 1

## Data Availability

All the data used during the study are available from the corresponding author on request.
