# Supplementary material for: TRAP1 inhibits MIC60 ubiquitination to mitigate the injury of cardiomyocytes and protect mitochondria in extracellular acidosis
Source: Cell Death Discov. 2021 Dec 14;7:389. doi: 10.1038/s41420-021-00786-5 (PMC8671480; doi:10.1038/s41420-021-00786-5)
Supplement: Supplementary file 1 — Supplemental figure legends [file 41420_2021_786_MOESM1_ESM.doc]

**Figure S1. Transgenic and acidosis rats model constructions.** (A) 100 ul lentiviral vectors (2×10^8^ TU/ml) were injected at 4-5 points on cardiac impulsing area using a 28-gauge needle (about 20 ul per site). (B) The pH values of arterial blood were detected using blood gas analyzer (ABL80FLEX, Radiometer Medica). (C) IHC assay was used to identify the transfection efficiency of TRAP1 and MIC60. Data are the means±SD from 6 rats. Group comparisons were performed by one-way analysis of variance followed by Tukey’s post hoc test. ^&^P<0.05 vs. Normal group.

**Figure S2. TRAP1 specifically interacted with and regulated MIC60 ubiquitination in in extracellular acidosis.** (A) CO-IP assays were used to detect the interaction of TRAP1 with MIC60, Prohibitin VDAC and TOM20. (B) The protein levels of Prohibitin, VDAC AND TOM20 were detected in pH 7.4 or pH 6.5. (C) Western blot was used to detect MIC60 protein ubiquitination. Sh-MIC60 and IgG group were as negative and blank control. Data are the means±SD from three independent experiments. Group comparisons were performed by one-way analysis of variance followed by Tukey’s post hoc test. NS: no statistically significant difference between groups. ^&^P<0.05 vs. 0 h. **^#^**P<0.05 vs. ov-Con group. *P<0.05 vs. sh-Con group.
