# Supplementary figures and images for: TRAP1 inhibits MIC60 ubiquitination to mitigate the injury of cardiomyocytes and protect mitochondria in extracellular acidosis
Source: Cell Death Discov. 2021 Dec 14;7:389. doi: 10.1038/s41420-021-00786-5 (PMC8671480; doi:10.1038/s41420-021-00786-5)

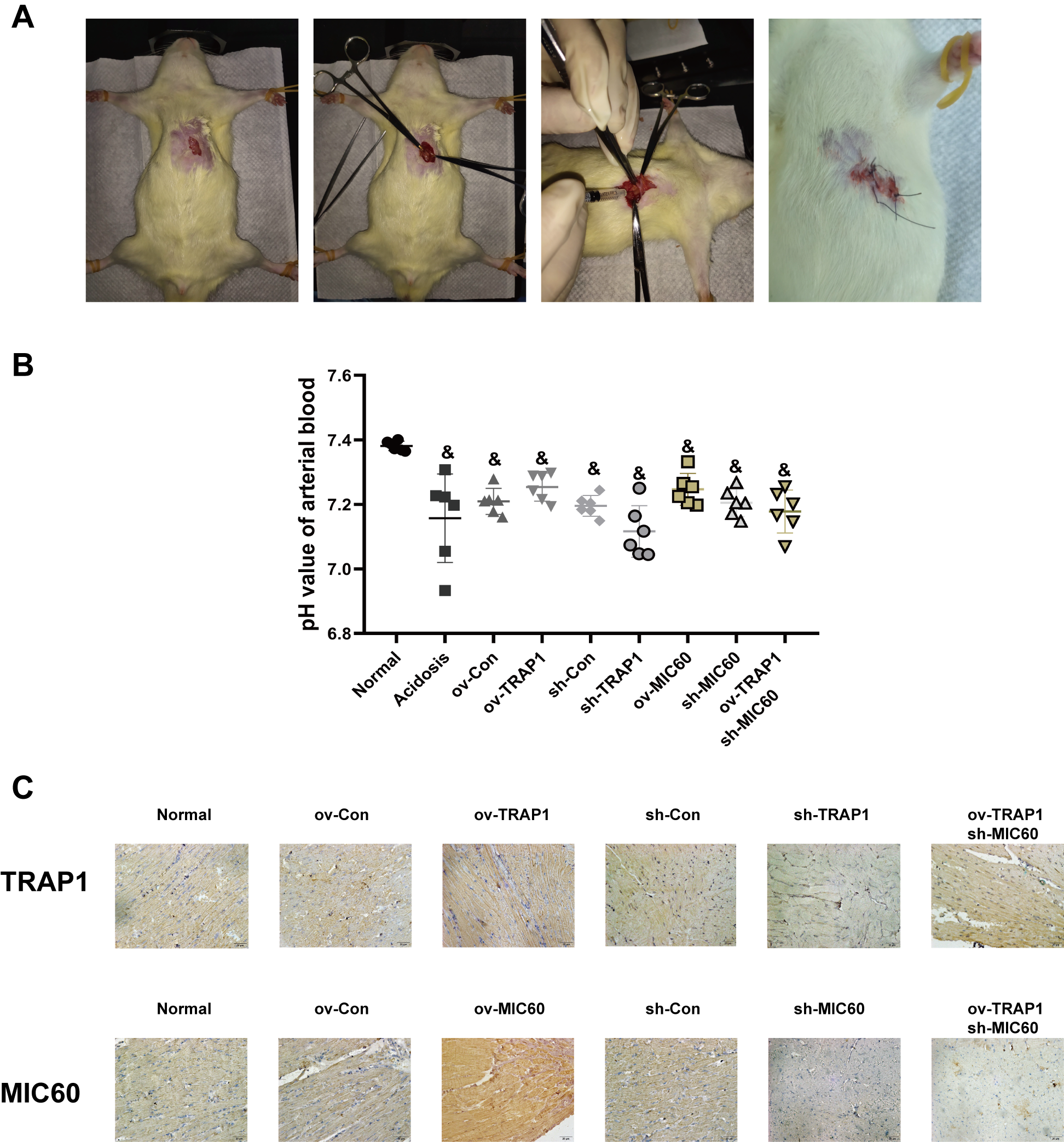

Supplement: Supplementary file 2 — Figure S1 [file 41420_2021_786_MOESM2_ESM.tif]

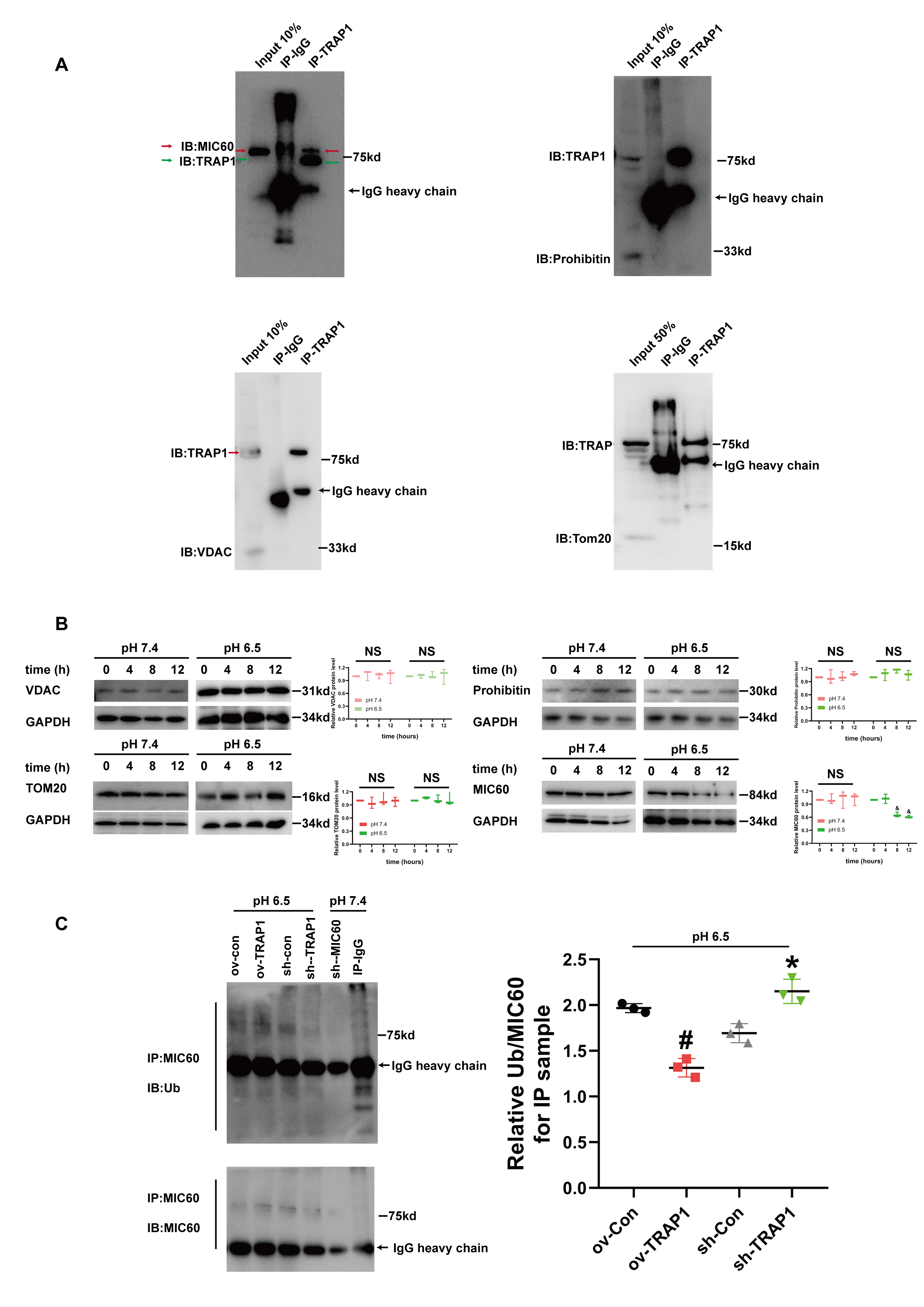

Supplement: Supplementary file 3 — Figure S2 [file 41420_2021_786_MOESM3_ESM.tif]
